# Optimization of combined temozolomide and peptide receptor radionuclide therapy (PRRT) in mice after multimodality molecular imaging studies

**DOI:** 10.1186/s13550-015-0142-y

**Published:** 2015-11-09

**Authors:** Sander M. Bison, Joost C. Haeck, K. Bol, S. J. Koelewijn, H. C. Groen, M. Melis, J. F. Veenland, M. R. Bernsen, M. de Jong

**Affiliations:** Department of Nuclear Medicine, Erasmus MC, Postbus 2040, Rotterdam, 3000, CA The Netherlands; Department of Radiology, Erasmus MC, Postbus 2040, Rotterdam, 3000, CA The Netherlands; Department of Medical Informatics, Erasmus MC, Postbus 2040, Rotterdam, 3000, CA The Netherlands

**Keywords:** NETs, PRRT, Temozolomide, Combination therapy, Multimodality imaging, Tumour perfusion

## Abstract

**Background:**

Successful treatments of patients with somatostatin receptor (SSTR)-overexpressing neuroendocrine tumours (NET) comprise somatostatin-analogue lutetium-177-labelled octreotate (^177^Lu-TATE) treatment, also referred to as peptide receptor radionuclide therapy (PRRT), and temozolomide (TMZ) treatment. Their combination might result in additive effects. Using MRI and SPECT/CT, we studied tumour characteristics and therapeutic responses after different (combined) administration schemes in a murine tumour model in order to identify the optimal treatment schedule for PRRT plus TMZ.

**Methods:**

We performed molecular imaging studies in mice bearing SSTR-expressing H69 (humane small cell lung cancer) tumours after single intravenous (i.v.) administration of 30 MBq ^177^Lu-TATE or TMZ (oral 50 mg/kg daily for 14 days). Tumour perfusion was evaluated weekly by dynamic contrast-enhanced MRI (DCE-MRI), whereas tumour uptake of ^111^In-octreotide was quantified using SPECT/CT until day 39 after treatment. Based on these results, seven different ^177^Lu-octreotate and TMZ combination schemes were evaluated for therapy response, varying the order and time interval of the two therapies and compared with single treatments.

**Results:**

PRRT and TMZ both resulted in tumour size reduction, accompanied by significant changes in MRI characteristics such as an enhanced tumour perfusion. Moreover, TMZ treatment also resulted in increased uptake of the SST analogue ^111^In-octreotide until day 13. In the subsequent therapy study, 90 % of animals receiving ^177^Lu-TATE at day 14 after TMZ treatment showed complete response, being the best anti-tumour results among groups.

**Conclusions:**

Molecular imaging studies indicated that PRRT after TMZ treatment could induce optimal therapeutic effects because of enhanced tumour uptake of radioactivity after TMZ, which was confirmed by therapy responses. Therefore, clinical translation of TMZ treatment prior to PRRT might increase tumour responses in NET patients as well.

## Background

Systemic internal radiation therapy using radiolabelled peptides, specifically targeting receptors overexpressed on tumour cells, is an attractive cancer treatment. A high absorbed radiation dose is being delivered to tumours with a tolerable toxicity in normal non-targeted tissues. This so-called peptide receptor radionuclide therapy (PRRT) has been shown to be a most effective treatment option for patients with SSTR-expressing neuroendocrine tumours (NETs) [1–3].

Most NETs are slowly proliferating tumours, making them relatively resistant to most chemotherapeutics. In more than 50 % of the patients, NETs are diagnosed at a relatively late stage, often with metastatic spread [4], which leaves little chance for curative surgery. Then, PRRT with radiolabelled SST analogues appeared to be an attractive cancer treatment. A high absorbed dose is being delivered to tumours with a tolerable toxicity in normal non-targeted tissues [1–3]. Radiolabelled SST analogues, such as ^111^In-DTPA-octreotide (^111^In-octreotide) or ^177^Lu-DOTA,Tyr^3^-octreotate (^177^Lu-TATE), show a high binding affinity for the somatostatin receptor type 2 (SSTR2), which is overexpressed on the vast majority of NETs [5].

PRRT has proven to be an effective therapy in most patients with NETs [1, 6]. Nevertheless, despite the improvement of progression-free survival and quality of life, complete remissions after PRRT are still rare. A combination of PRRT with radio-sensitizing chemotherapeutics, such as capecitabine (5-fluorouracil) or temozolomide (TMZ), might induce synergistic effects [7–9]. Currently, phase II clinical trials combining PRRT, using ^177^Lu-TATE and capecitabine or both capecitabine and TMZ, are ongoing. The first analyses showed promising results [8, 10].

When PRRT with chemotherapeutics are being combined, also potential negative interactions between the two therapies should be taken into consideration. Chemotherapy might affect the SSTR2 expression on tumour cells, on which PRRT depends for targeting of radionuclides, for example [11, 12]. Moreover, tumour vasculature might be affected by chemotherapy whereas efficient/adequate tumour perfusion is required for local delivery of ^177^Lu-TATE [13]. To obtain an optimal therapeutic scheme for the combination of PRRT and TMZ, information on the aforementioned tumour characteristics during treatment is required, which was the aim of this pre-clinical study using an H69-tumour-bearing mouse model. Tumour perfusion was monitored by dynamic contrast-enhanced (DCE)-MRI and SSTR expression by ^111^In-octreotide SPECT. The results from these imaging studies were applied in the design of different therapeutic schemes to stratify maximum therapeutic efficacy using TMZ and PRRT.

## Methods

### Experimental setup

We have started a pilot study in which H69 tumour-bearing mice were treated with increasing amounts of ^177^Lu-TATE to determine which dose resulted in clear tumour responses but without complete cure to allow detection of additional effects during combination therapy. Three groups (*n* = 7) of H69-tumour-bearing mice were treated with 10, 20 or 30 MBq ^177^Lu-TATE and compared with untreated control animals. Monitoring of body weight and tumour size was performed until day 28 after the start of treatment.

For TMZ treatment, different dosing schedules have been reported, including a total administered dose of >1000 mg/kg [14] or the administration of one or more cycles during 5 days, in which a dose of 50 or 120 mg/kg/day was administered [15, 16]. Since we planned to administer only one cycle, we used a dose of 50 mg/kg/day for 2 weeks, to study the effects of TMZ treatment in the H69 model, supplemented with investigations into combination of PRRT and TMZ. In this study, the earlier determined optimal dose of 30 MBq ^177^Lu-TATE was used.

#### Imaging studies to determine tumour characteristics during PRRT or TMZ treatment

**Fig. 1:**
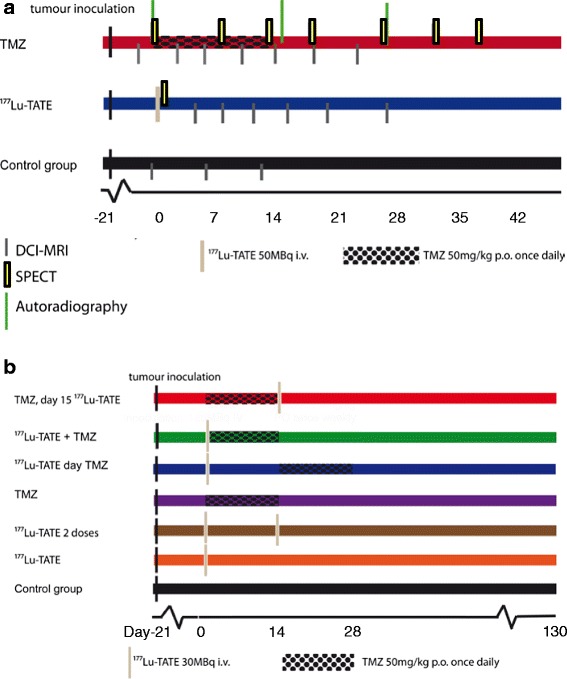
Timeline for the imaging study (**a**) and for the combination therapy studies (**b**)

Three groups of mice were included in this imaging study (Table 1). The PRRT group received a single dose of 50 MBq ^177^Lu-TATE on day 1, and the TMZ group was treated with 50 mg/kg TMZ administered orally for 2 weeks, starting at day 0. The control group received saline. The timeline for these studies is shown in Fig. 1a.

**Table 1:** Treatment and imaging for each group

Group	Number of mice	Treatment	Imaging
^177^Lu-TATE	4	50 MBq ^177^Lu-TATE i.v.	6 × MRI, 1 × SPECT/CT
TMZ	7	TMZ 50 mg/kg p.o. for 14 days	7 × MRI, 7 × SPECT/CT
Control	3	Saline	3 × MRI

^*177*^ *Lu-TATE* lutetium-177-labelled octreotate, *TMZ* temozolomide

##### PRRT group

Mice (*n* = 4) received a baseline DCE-MRI scan 1 day prior to administration of 50 MBq ^177^Lu-TATE. During follow-up of the therapy, MRI scans were acquired on days 4, 7, 11, 15, 20 and 28. Moreover, a SPECT/CT scan to determine the tumour uptake of ^177^Lu-TATE was performed 24 h after administration.

##### TMZ group

Mice (*n* = 7) received one baseline DCE-MRI scan 2 days prior start of TMZ treatment, which was repeated once weekly during and after TMZ treatment until day 28. To determine the level of radiopeptides uptake, ^111^In-octreotide SPECT/CT scanning was performed 1 day prior start of TMZ treatment and repeated once weekly for 6 weeks during and after TMZ. At day 1 (before TMZ), day 14 (after TMZ) and day 28, when tumours showed maximal response, three mice where sacrificed to collect the H69 xenograft to determine the level of SSTR expression by in *vitro* autoradiography using ^111^In-octreotide.

##### Control group

Non-treated mice (*n* = 3) received four DCE-MRI scans: at days 1, 5 and 12.

#### Combination therapy of PRRT and TMZ

Seven groups of mice were included to compare H69 responses after different combination treatment schedules, including a placebo control group (receiving oraplus solution without TMZ p.o. once daily.) (Table 2). Uptake of ^177^Lu-TATE was quantified in three to six tumours of each group after PRRT by SPECT imaging. The timeline for these studies is shown in Fig. 1b.

**Table 2:** Treatment schedule and number of mice for each group

Group	Treatment		Number of mice
	^177^Lu-TATE	TMZ	
Control	–	Placebo	5
1: PRRT single	30 MBq day 0	Placebo	8
2: PRRT double	30 MBq day 0	Placebo	8
3: TMZ	–	50 mg/kg for 14 days from day 0	8
4: PRRT + TMZ at day 14	30 MBq day 0	50 mg/kg for 14 days from day 14	8
5: PRRT + TMZ	30 MBq day 0	50 mg/kg for 14 days from day 0	8
6: TMZ + PRRT at day 14	30 MBq day 14	50 mg/kg for 14 days from day 0	10

^*177*^ *Lu-TATE* lutetium-177-labelled octreotate, *TMZ* temozolomide, *PRRT* peptide receptor radionuclide therapy

### Tumour cell line

The SSTR2-expressing human small cell lung cancer cell line H69 was obtained from ECACC (Salisbury, UK) and grown in RPMI medium (Gibco, Invitrogen Corp., Breda, The Netherlands) supplemented with 10 % heat-inactivated foetal bovine serum.

### Animals and tumour model

All animal experiments have been conducted with prior approval of the animal ethics committee of our institution and performed in accordance with Dutch laws. Male NMRI nu/nu mice (body weight ~33 g) were obtained from Harlan (Heerlen, the Netherlands). One week after arrival, at the age of 5–7 weeks, mice were inoculated subcutaneously with 10^7^ H69 cells in 0.2-ml HBSS. For all experiments, animals were randomized into matching treatment groups regarding tumour size at the start of treatment 4 weeks after tumour inoculation. Randomized treatment groups were created by matched pairs randomization. Three times a week, mice were weighed and length and width of the tumour were measured using a calliper by a person blinded for the treatment. Tumour volume was calculated using the formula 0.5(length × width)^1.5^ adapted from the SWOG criteria. Mice were euthanized when >10 % loss of body weight (BW) since start of the experiment was observed or when tumour volume exceeded 1800 mm^3^.

### Chemotherapeutics

Temozolomide was obtained from Sun Pharmaceutical Industries Europe B.V. (Hoofddorp, The Netherlands). In the pilot study, TMZ was dissolved as 8 mg/ml 50 % glucose jelly and 200 μl was administered orally. For the imaging and final therapy studies, a 8 mg/ml solution TMZ was prepared in Oraplus (Paddock laboratories, Inc. Minneapolis USA) and 200 μl aliquots were administered by oral gavage 5 days a week for 2 weeks resulting in a dose of 50 mg/kg/day TMZ.

### Radionuclides and peptides

DOTA,Tyr^3^-octreotate was obtained from Mallinckrodt, St Louis, MO, and ^177^LuCl_3_ was obtained from NRG Petten, The Netherlands. ^177^Lu-TATE was prepared as described previously [17] with a specific activity of 100 MBq/2.75 μg peptide, and 10, 20, 30 or 50 MBq was injected intravenously (i.v.) in a volume of 200 μl via the tail vein. Labelling of ^111^In-DTPA-octreotide (OctreoScan, Covidien, Petten, The Netherlands) was performed as described at a specific activity of 30 MBq/1.0 μg DTPA-octreotide [17].

### SPECT/CT

During scanning experiments, 2.0 % isoflurane/O_2_ gas anaesthesia was applied at 0.5 ml/min. Twenty-four hours after injection of ^177^Lu-TATE or ^111^In-octreotide, helical SPECT/CT of the tumour region was performed with a four-headed NanoSPECT/CT system (BioScan, Washington DC USA) with nine pinhole mice collimators (diameter 1.4 mm) per head. The scans were obtained using 24 projections of 120 s per projection and a quality factor of 0.7. SPECT scans were reconstructed iteratively on a 256 × 256 matrix, using HiSPECT NG software (Scivis, GmbH Göttingen Germany) and ordered subset expectation maximization (OSEM). The total amount of radioactivity (MBq) in the tumour was quantified by 3D quantification using InVivoScope software (IVS, Bioscan, Washington DC USA). To achieve accurate quantification, the camera was calibrated by scanning a 20-mL polypropylene tube phantom filled with a known amount of ^177^Lu or ^111^In radioactivity. During scanning, body temperature of the mice was maintained using a heated bed.

### MRI

Imaging was performed on a pre-clinical 7.0T scanner (Discovery MR901, Agilent technologies/GE Healthcare) with the standard imaging gradient set (300 mT/m, slew rate of 1000 mT/m/ms—rise time = 300 μs). A 150-mm transmit coil and four-channel surface coil (5 cm FOV) were used to acquire all images.

DCE-MRI data were acquired following a bolus i.v. injection of Gadobutrol (Gadovist; Bayer Healthcare, Germany). The acquisition parameters used were repetition time (TR) = 10 ms, echo time (TE) = 2 ms, flip angle 12°, matrix size = 116 × 116 and slice thickness = 0.8 mm with a total imaging volume of 50 × 50 × 32 mm^3^ (x, y, z). Four k-space segments were acquired using a segmented readout sequence called TRICKS [18]. This resulted in a temporal resolution of 4.7 s. Before contrast administration, four to five time points were acquired to obtain the baseline signal intensity. Prior to acquiring the DCE-MRI image series, a saturation recovery T1 map was acquired using a spin echo sequence with varying TRs, settings: TE = 8 ms, TR = 200, 400, 800 and 1600 ms. T2 maps were also acquired with a spin echo sequence varying TE, settings: TR = 1200 ms, TE = 8, 16, 25 and 35 ms. All mapping images were acquired with slice thickness 0.8 mm, 5 cm FOV and 256 × 256 matrix.

### Image analysis

Analysis of the SPECT data: the concentration of activity in the tumours was calculated by dividing the total amount of activity in the tumour determined by 3D quantification with HiSPECT NG software through the 3D tumour volume determined during this quantification.

For analysis of the DCE-MRI data, we used two methods: semi-quantitative and quantitative analysis. All calculations were performed using Matlab (Mathworks co.). In semi-quantitative analysis, the signal-intensity time curves were used to determine the time to peak (TTP), maximum signal enhancement (Smax) and the area under the curve (AUC) for the total curve and also for the first 60 s (AUC60). The AUC and AUC60 were calculated using a triangulation algorithm. Furthermore, the wash-in was calculated as a linear slope from the first point before contrast enhancement to the first maximum. The wash-out was calculated from the slope of the first maximum to the measurement at 60 s after injection. The wash-out curve was not necessarily linear in shape, but using this method, we were able to discriminate the wide variety in wash-out kinetics. In order to calculate quantitative parameters from the DCE-MRI data, the signal-intensity time curves were converted to contrast-concentration time curves using a T1-map calibration. A pharmacokinetic compartment model could be fitted to these contrast-concentration time curves. Quantitative analysis consisted of calculation of k^trans^ and k_ep_, calculated according to Tofts perfusion model [19]. A population-based average arterial input function (AIF) obtained from Weidensteiner [20] was used for consistency in the results. For the response to therapy, comparison of the tumour perfusion parameters was performed; regions of interest were drawn around the tumours, and mean parameter values were calculated. k^trans^ is the calculated rate constant for the passage of contrast agent into the tissue. The rate constant k_ep_ is the reflux of contrast agent from tissue back to the blood. The results presented show the k^trans^ which performed slightly better than the k_ep_ in this model due to the limited amount of wash-out in the 10-min scantime.

### In *vitro* autoradiography on tumours treated with TMZ during the imaging study

Sections of 10 μm were sliced from frozen tumours (Cryo-Star HM 560 M; Microm, Walldorf, Germany), mounted on Superfrost plus slides (Menzel, Braunschweig, Germany) and incubated with 10^−9^M ^111^In-octreotide with and without an excess (10^−6^M) of unlabelled octreotide. Adjacent sections were stained with haematoxylin/eosin. Tumour sections were exposed to SR phosphor imaging screens (Packard Instruments Co., Meriden, USA) in X-ray cassettes. After 48-h exposure, screens were read by a Cyclone phosphor imager and analysed using OptiQuant 03.00 (Perkin Elmer, Groningen, The Netherlands). Autoradiogram quantification was expressed in density light units (DLU) per square millimetre.

### Statistics

Prism software version 5.0 (Graph Pad) was used to analyse tumour growth and determine statistical significance between groups. One-way ANOVA was used for statistical analysis of tumour uptake, and results are given as mean ± SD. A log rank test was performed for curve comparison.

## Results

### ^177^Lu-TATE dose-response study

**Fig. 2:**
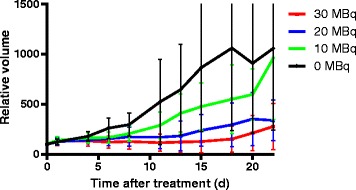
Dose-finding study: H69 tumour volume as a percentage compared with day 0 (±SD) after treatment with different amounts of ^177^Lu-TATE, seven mice/group

All three ^177^Lu-TATE groups showed delay of tumour growth compared with the control group (Fig. 2). As expected, the most significant tumour growth suppression was found in the 30 MBq group, although complete response was not reached in any mouse.

### Imaging studies

#### Effect of single-agent treatment on tumour growth

**Fig. 3:**
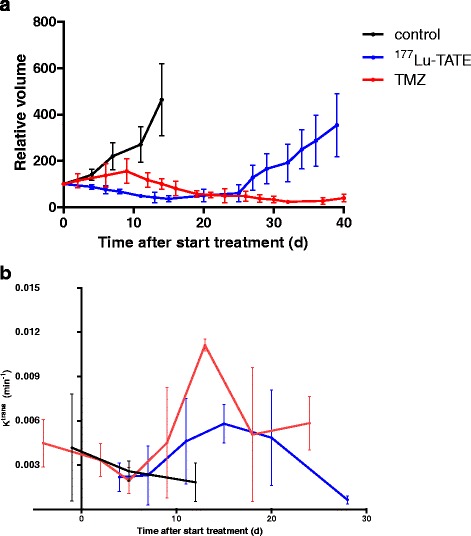
Effect on H69 tumour volume (**a**) and tumour perfusion (**b**) of single-agent treatment. **a** Tumour volume as a percentage compared to day 0 ± SD. **b** Average intra-tumoural k^trans^ was calculated, and the presented data show the average across tumours with the SD. The control group (*n* = 3) (*black line*) received saline, the PRRT group (*n* = 4) was treated with 50 MBq ^177^Lu-TATE (*blue line*) on day 0, and the temozolomide (TMZ) group (*n* = 7) (*red line*) was treated for 14 days with orally administered TMZ at a dose of 50 mg/kg/day

Treatment with either ^177^Lu-TATE or TMZ resulted in transient reduction in tumour size/volume, confirming previous results. However, the kinetics of the tumour response after the two treatments differed (Fig. 3a).

The average ^177^Lu uptake in the four tumours in the PRRT group was 1.7 ± 0.1 kBq/mm^3^ (3.4 ± 0.2 %IA/g) at 24 h after administration of 50 MBq ^177^Lu-TATE. Maximum reduction of tumour volume was reached at ~day 14, after which the tumours increased in size again. After an average of 43 days, euthanasia was needed because the maximal allowed tumour size (1800 mm^3^) was reached.

In mice treated with TMZ, the tumour volumes increased until day 9 reaching maximum tumour sizes, after which decrease of tumour sizes was observed. At day 51, at the end of the imaging study, the average tumour volume increased again; however, at that time, none of the tumours exceeded a volume of 1800 mm^3^.

#### Tumour perfusion during ^177^Lu-TATE or TMZ treatment

Tumours subjected to TMZ and ^177^Lu-TATE initially showed a fast decrease in k^trans^, reaching minimum values at day 4 ((2.2 ± 2) × 10^−3^ min^−1^ for ^177^Lu-TATE) or day 5 ((2.0 ± 2) × 10^−3^ min^−1^ for TMZ). When tumour size decreased, an increase in k^trans^ was measured. The local maximum in k^trans^ values was reached approximately 2 weeks after (start of) treatment. Tumours in the group receiving TMZ showed the highest k^trans^ values at day 13 ((11 ± 1) × 10^−3^ min^−1^); the ^177^Lu-TATE group’s maximum k^trans^ value was reached at day 15 ((5.8 ± 2) × 10^−3^ min^−1^). During further follow-up, the k^trans^ values decreased again in both the TMZ and ^177^Lu-TATE group.

#### SSTR2 expression and uptake of ^111^In-octreotide before and after TMZ treatment

**Fig. 4:**
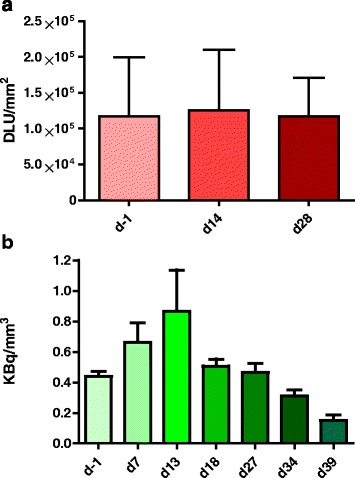
Graphs showing SSTR expression of H69 tumours treated with temozolomide (TMZ) and tumour uptake of ^111^In-octreotide. **a** Quantification of somatostatin receptor (SSTR) density in frozen sections of H69 tumours as determined by ^111^In-octreotide in *vitro* autoradiography. The amount of radioactivity is expressed in density light units (DLU) per square millimetre. SSTR expression was quantified at day 0, day 14 (1 day after TMZ treatment), and day 28 when tumours reached a minimal volume after TMZ treatment. Three tumours/day were examined. **b** Based on SPECT images, the average tumour concentration of ^111^In-octreotide was determined 24 h after administration. Mice were treated with TMZ from day 0 until day 13

In *vitro* autoradiography results showed no difference in SSTR2 expression of H69 xenografts before (day 0) or after (day 15 and day 28) TMZ treatment (Fig. 4a). Yet, SPECT imaging showed that at day 0 (average tumour volume 456 mm^3^), the average radioactivity after injection of ^111^In-octreotide was 0.4 ± 0.2 kBq/mm^3^ (1.5 ± 0.4 %IA/g) H69 xenograft, while after 14 days of TMZ treatment, this was increased until 0.9 ± 0.3 kBq/mm^3^ (2.9 ± 0.9 %IA/g) (average tumour volume 442 mm^3^). When the administration of TMZ was discontinued, the ^111^In-H69 uptake dropped again to 0.5 ± 0.2 kBq/mm^3^ (1.7 ± 0.5 %IA/g) at day 18 and 0.5 ± 0.2 kBq/mm^3^ (1,5 ± 0.5 %IA/g) at day 27, further declining until day 39 (Fig. 4b).

### Therapy study

#### Responses to the different treatment schedules

**Fig. 5:**
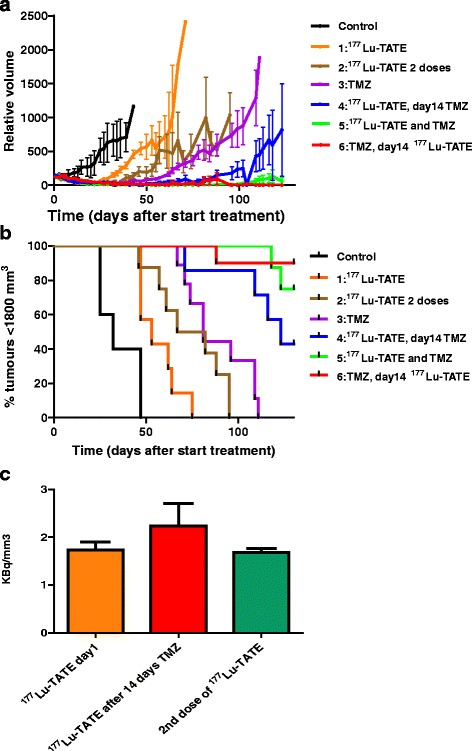
Graphs showing for each treatment group **a** average H69 tumour volume, **b** percentage of animals with tumours <1800 mm^3^, and **c** average tumour radioactivity at 24 h after administration of ^177^Lu-TATE A. Average tumour volume as a percentage compared with day 0. Tumours >1800 were taken out of the follow-up, which influenced the average tumour volume shown in the curves. The control group was treated with placebo, 30 MBq ^177^Lu-TATE was i.v. administered at day 0 to mice in groups 1, 4 and 5, at day 14 for group 6 and both at day 0 and day 14 for group 2. TMZ was administered orally once daily for 14 days at a dose of 50 mg/kg from day 0 to mice in groups 3, 4 and 6, starting at day 14 for group 5. Eight to ten mice/group. **c** Average tumour radioactivity: *orange bar*: groups 1, 2 (first dose), 4, 5; *red bar*: group 6; *green bar*: group 2 (second dose). Numbers, respectively, 14, 6 and 6 mice/group

As expected, all mice in the control group had to be euthanized due to excessive tumour growth. This was also true for the mice in the single-treatment groups with ^177^Lu-TATE (groups 1 and 2) or TMZ (group 3), albeit with a significant delay of 20–50 days (Fig. 5b). The median survival time (MST) to reach the maximum tumour size of 1800 mm^3^ was 32 days for the mice in the control group versus 53 days for the single ^177^Lu-TATE group 1 and 74 days for the double ^177^Lu-TATE group 2. The single TMZ group 3 reached an extended MST of 81 days.

In all three groups treated with a combination of ^177^Lu-TATE and TMZ, an additive effect of the two treatments was determined. Since in several mice complete tumour response was reached, MST could not be determined. Regarding survival, the difference between the best performing single-therapy group 3 (TMZ) and group 4 (TMZ 14 days after ^177^Lu-TATE) was significant (*p* = 0.046), whereas the other combination groups performed even better. Thus, the applied treatment schedules in these three groups had a striking impact on tumour response. In group 4, still 57 % of tumours reached a volume of 1800 mm^3^ before the end of the study at day 123. When ^177^Lu-TATE and TMZ were combined from the start (group 5), only 25 % of tumours reached this maximum size. By far, the best result was obtained in group 6 with ^177^Lu-TATE administered after 14 days TMZ treatment. Only one tumour (10 %) escaped from treatment, whereas the other tumours showed complete response.

#### Tumour uptake of ^177^Lu-TATE

The uptake of ^177^Lu-TATE in H69 tumours was quantified 24 h after administration of 30 MBq ^177^Lu-TATE based on SPECT/CT imaging. As reference, the average ^177^Lu-tumour uptake was 1.7 ± 0.2 kBq/mm^3^ without previous treatment. When after 14 days a second dose of ^177^Lu-TATE was given, a comparable ^177^Lu-tumour concentration of 1.7 ± 0.1 kBq/mm^3^ (5.6 ± 0.3 %IA/g) was found. However, in mice receiving ^177^Lu-TATE on day 14 after initial TMZ for 14 days, a significantly higher uptake of 2.2 ± 0.5 kBq/mm^3^ (7.5 ± 1.5 %) was determined (*p* = 0.013) (Fig. 5c).

## Discussion

Despite the fact that significant tumour response rates have been reported after treatment of NET patients with ^177^Lu-TATE or [90Y-DOTA]-D-Phe1-Tyr3-octreotide (90Y-DOTATOC) [21, 22], complete responses are still rare, urging for improvements of PPRT protocols. TMZ is currently being applied in combination with radiotherapy for treatment of glioblastoma patients [23]. TMZ (+ cap) is also effective in NET patients [24, 25]. Therefore, we examined in mice if this alkylating agent offered synergistic effects when combined with ^177^Lu-TATE PRRT.

In this pre-clinical study, we aimed to elucidate the most effective treatment schedule of the TMZ and PRRT combination. We applied a TMZ dose of 50 mg/kg daily for 2 weeks, which appeared to be an effective dose in our H69 model not resulting in complete responses. The same was true for a dose of 30 MBq ^177^Lu-TATE leading to a significant delay of H69-tumour size increase.

The human small cell lung cancer (SCLC) H69 cell line, used in our studies, expresses SSTR2 in high densities. As a subtype of NETs, SCLC accounts for about 20 % of all lung cancers [26] and shows a 5-year survival rate of only 20 % [26]. For this type of cancer, there is a clear need to find therapeutic methods resulting in better survival. Several chemotherapeutics, including TMZ, are being used to treat SCLC [27], making the H69 cell line a suitable model for pre-clinical in vivo PRRT research using radiolabelled SST analogues [28]. To study dosimetry and tumour response, Schmitt et al. applied several activity doses of ^177^Lu-TATE for PRRT of H69 tumour-bearing mice [28, 29]. In their study, tumours showed a significantly higher uptake of radioactivity compared with all normal organs [28]. In a therapy study, using substantially larger tumours than in our study (respectively, 1000 and 2000 mm^3^ at day 0), increasing growth delays using doses of 45, 60 and 120 MBq or two fractions of 45 MBq ^177^Lu-TATE were found [29].

In the clinical trials in which PRRT was combined with capecitabine and TMZ, the focus mainly has been on the radio-sensitizing effects of the chemotherapeutics [8, 30], not considering other effects these agents might have on tumour characteristics. In our opinion, the potential influence of chemotherapeutics on tumour perfusion and SSTR2 expression is of major importance as well, to determine an optimal scheme for the combination of PRRT with chemotherapeutics.

In this study, we measured an increase of H69-tumour perfusion after TMZ treatment. The highest values after 14 days of TMZ treatment coincide with a peak in the uptake of radiolabelled octreotide. The latter observation was confirmed during therapy, as ^177^Lu-uptake in H69 tumours after ^177^Lu-TATE administration was significantly higher when mice were pre-treated for 14 days with TMZ. This was not the case when pre-treatment consisted of an additional dose of ^177^Lu-TATE.

This increased tumour uptake/retention of radioactivity after TMZ treatment can be explained by different phenomena. A relation between tumour volume and concentration of radioactivity has been described, indicating an increased concentration of radioactivity in smaller tumours [28]. In our studies, however, the average tumour size was in the same range at day 0 and at day 13, respectively, a mean tumour volume of 456 ± 122 versus 442 ± 66 mm^3^, demonstrating that the peak of radioactivity uptake at day 13 was not influenced by tumour volume. At later time points, when tumour sizes continued to decrease as a result of TMZ treatment, also the concentration of ^111^In-octreotide decreased.

An increased level of SSTR2 expression might also explain the peak of radiopeptide uptake after 14 days of TMZ. Using in *vitro* studies, Fueger et al. showed increased SSTR2 expression in pancreatic tumour cells 4 days after exposure to several chemotherapeutics, not including TMZ [11, 12]. This phenomenon could not explain our findings in the current study, however, as we found no differences in SSTR2 expression prior to or after TMZ treatment, when in vivo H69-uptake of ^111^In-octreotide peaked.

Therefore, increased tumour perfusion caused by TMZ treatment offers the best explanation for the raised levels of ^111^In and ^177^Lu tumour uptake, as was explored and confirmed by application of measurement of perfusion parameters by DCE-MRI.

In multiple studies, TMZ, when administered in a metronomic (repetitive, low dose) schedule, has been demonstrated to have anti-angiogenic effects that may result in normalized tumour vasculature depending on the administered TMZ dose [31, 32]. Therefore, direct or indirect anti-angiogenic effect of chemotherapeutics can improve tumour perfusion and reduce the interstitial pressure [33], enabling enhanced delivery to the tumour of therapeutic compounds, including radiolabelled peptides. Our measurements with DCE-MRI support the suggestion that there is an important relationship between functional vasculature and radiopeptide uptake [34].

Considering the anti-tumour response, a small but not significant (*p* = 0.28) difference was found between group 5 (concurrent ^177^Lu-TATE and TMZ) and group 6 (^177^Lu-TATE after 14 days TMZ treatment) regarding CR, with 75 versus 90 % CR, respectively. However, when we compare group 6 with group 4 (the group in which ^177^Lu-TATE was administered at day 1 and TMZ treatment started at day 14), a striking and significant difference (*p* = 0.046) between those groups was found, in agreement with the fact that TMZ potentiates the effect of ^177^Lu-TATE.

Therefore, the optimal response found in the group receiving ^177^Lu-TATE after TMZ treatment might be based on a combination of factors:

1. Increased uptake of ^177^Lu-TATE because of enhanced perfusion resulting in a higher absorbed tumour radiation dose and therefore an increased anti-tumour response [35].
2. Increased radiosensitivity induced by TMZ. The cytotoxicity of TMZ is primarily due to alkylation at the *O*6 position of guanine combined with an additional alkylation at the *N*7 position. TMZ has been reported to act synergistically with radiation therapy, as alkylated guanine has been reported to be radio-sensitizing [9], as was shown in multiple types of cancer cells [9, 36]. As the half-life of ^177^Lu is 6.7 days [28], also the group receiving ^177^Lu-TATE at the same day that TMZ treatment was started might have had some benefit from the radio-sensitizing effects of TMZ.
3. Increased oxygenation might have contributed to improved therapeutic responses, as was demonstrated for radiation therapy [37]. After beta-particle radiation, DNA damage occurs as a result of the creation of free oxygen radicals [38], so the effect of ^177^Lu-TATE treatment might be enhanced when tumour oxygenation is improved. The mice receiving ^177^Lu-TATE 14 days prior to TMZ treatment did not have this benefit.

Despite the fact that our model has been carefully chosen, it has important differences from the patient situation. Differences in, e.g. tumour growth rate might influence the outcome of these studies in a clinical setting. Therefore, the translational value of our findings into the clinic remains to be proven.

During our combination therapy study, no signs of severe toxicity like, e.g. remarkable loss of weight have occurred. Nevertheless, during PRRT as well as chemotherapy, toxicity is an aspect of great concern. In a clinical study, a treatment scheme in which PRRT was combined with capacetabine and TMZ has been shown to be safe [8]. Considering increased uptake of radionuclides after TMZ treatment, in our opinion, this is likely to be solely applicable to the tumour because of its immature vasculature. However, further investigation on toxicity and uptake of radionuclides by the normal organs is warranted.

## Conclusions

The use of SPECT/CT and MRI enabled us to optimize a treatment schedule for the combination of PRRT and TMZ in H69-xenografted nude mice. Increased perfusion following TMZ pre-treatment, determined by DCE-MRI, coinciding with increased uptake of ^177^Lu-TATE as proven by SPECT/CT, was reflected by the induced therapeutic effects we observed.

## Compliance with ethical standards

### Ethical approval

All applicable international, national and/or institutional guidelines for the care and use of animals were followed.
